# Quantifying Killing of Orangutans and Human-Orangutan Conflict in Kalimantan, Indonesia

**DOI:** 10.1371/journal.pone.0027491

**Published:** 2011-11-11

**Authors:** Erik Meijaard, Damayanti Buchori, Yokyok Hadiprakarsa, Sri Suci Utami-Atmoko, Anton Nurcahyo, Albertus Tjiu, Didik Prasetyo, Nardiyono, Lenny Christie, Marc Ancrenaz, Firman Abadi, I Nyoman Gede Antoni, Dedy Armayadi, Adi Dinato, Ella, Pajar Gumelar, Tito P. Indrawan, Kussaritano, Cecep Munajat, C. Wawan Puji Priyono, Yadi Purwanto, Dewi Puspitasari, M. Syukur Wahyu Putra, Abdi Rahmat, Harri Ramadani, Jim Sammy, Dedi Siswanto, Muhammad Syamsuri, Noviar Andayani, Huanhuan Wu, Jessie Anne Wells, Kerrie Mengersen

**Affiliations:** 1 People and Nature Consulting International, Jakarta, Indonesia; 2 School of Biological Sciences, The University of Queensland, Brisbane, Australia; 3 School of Archaeology and Anthropology, Australian National University, Canberra, Australia; 4 The Nature Conservancy – Indonesia Forest Program, Jakarta, and Bogor, Indonesia; 5 Department of Plant Protection, Faculty of Agriculture, Institut Pertanian Bogor, Bogor, Indonesia; 6 The Indonesian Association of Primatologists (PERHAPPI), Bogor, Indonesia; 7 Faculty of Biology, Universitas Nasional, Jakarta, Indonesia; 8 Borneo Orangutan Survival Foundation, Orangutan Reintroduction Program Central Kalimantan, Palangkaraya, Indonesia; 9 World Wide Fund for Nature Indonesia, Pontianak, Indonesia; 10 Kinabatangan Orang-utan Conservation Project, Sandakan, Sabah, Malaysia; 11 Borneo Ecology and Biodiversity Conservation Institute (BEBSiC), Samarinda, Indonesia; 12 Friends of the National Park Foundation (FNFP), Kumai, Indonesia; 13 People Resources and Conservation Foundation (PRCF), Pontianak, Indonesia; 14 SuaR Institute, Nanga Pinoh, Indonesia; 15 BIOMA Foundation, Samarinda, Indonesia; 16 Gunung Palung Orangutan Conservation Program, Ketapang, Indonesia; 17 Mitra Lingkungan Hidup KalTeng, Palangkaraya, Indonesia; 18 Orangutan Foundation International, Pangkalanbun, Indonesia; 19 Yayasan Dian Tama, Pontianak, Indonesia; 20 Simpur Hutan, Pontianak, Indonesia; 21 Yayasan Titian, Pontianak, Indonesia; 22 Yayasan Perhimpunan TeROPONG, Palangkaraya, Indonesia; 23 Sylva Indonesia PC.UNTAN, Pontianak, Indonesia; 24 Yayasan Riak Bumi, Pontianak, Indonesia; 25 Yayasan Cakrawala Indonesia, Palangkaraya, Indonesia; 26 Forum Komunikasi Kader Konservasi Indonesia (FK3I) Kalbar, Pontianak, Indonesia; 27 Wildlife Conservation Society, Bogor, Indonesia; 28 School of Mathematical Sciences, Queensland University of Technology, Brisbane, Australia; Zoological Society of London, United Kingdom

## Abstract

Human-orangutan conflict and hunting are thought to pose a serious threat to orangutan existence in Kalimantan, the Indonesian part of Borneo. No data existed prior to the present study to substantiate these threats. We investigated the rates, spatial distribution and causes of conflict and hunting through an interview-based survey in the orangutan's range in Kalimantan, Indonesia. Between April 2008 and September 2009, we interviewed 6983 respondents in 687 villages to obtain socio-economic information, assess knowledge of local wildlife in general and orangutan encounters specifically, and to query respondents about their knowledge on orangutan conflicts and killing, and relevant laws. This survey revealed estimated killing rates of between 750 and 1800 animals killed in the last year, and between 1950 and 3100 animals killed per year on average within the lifetime of the survey respondents. These killing rates are higher than previously thought and are high enough to pose a serious threat to the continued existence of orangutans in Kalimantan. Importantly, the study contributes to our understanding of the spatial variation in threats, and the underlying causes of those threats, which can be used to facilitate the development of targeted conservation management.

## Introduction

Effective wildlife and nature conservation requires balancing human development with the impacts this has on wildlife populations and their habitats [1]. The impacts are most severe where development is fuelled by exploitation of natural resources such as forests [2], [3]. Such situations are characteristic for many rapidly developing emerging economies in tropical Asia, Africa, and South America. In the forested parts of these regions, the conversion frontier from natural ecosystems into more intensively managed agro- and silvicultural lands is rapidly shifting; forests with few people and much wildlife are being replaced by human-dominated landscapes where few forest species survive. At the conversion frontier and in highly degraded forest areas, human-wildlife conflicts are common, because animals are being restricted into increasingly small forest fragments [4], [5], [6] and increasing human density adds further pressure in the forest transition zone. Even though it is commonly acknowledged in conservation that human-wildlife conflict can result in killing of animals, the relative scale of conflict and killing, as well as the underlying reasons for killings and the factors that influence them, are not well understood [7].

Orangutans (*Pongo* sp.) are a good example of an understudied species regarding hunting and agricultural conflict. Anecdotal information suggests that hunting of orangutans is common, at least in parts of their range [8], and hunting is thought to have been a main factor in historic population declines [9], [10]. Still, such studies are suggestive for present hunting levels only and there are no quantitative data on hunting that could support the hypothesis that hunting is currently a major factor in the decline of orangutan populations. Similarly, conservationists assume that people and orangutans clash over agricultural resources, sometimes resulting in orangutan killing, but the data to substantiate this assumption and quantify its impacts on the population are lacking [4], [11], [12]. Because both orangutan species (*P. pygmaeus* and *P. abelii*) are threatened with extinction in the wild [13], understanding the severity of different threats to these species, the spatial variation of those threats, and their underlying socio-cultural and ecological factors, are crucial for effective conservation.

This study aims to address the lack of quantitative information on human-induced orangutan mortality and human-orangutan conflicts and the socio-cultural and ecological factors that influence these factors. We conducted an interview-based survey among villagers living in the orangutan's range in Kalimantan, the Indonesian part of the island of Borneo. The objective of this particular paper is to understand the threats and the underlying drivers of orangutan killing in three Kalimantan provinces. We specifically focus on the role of conflict between orangutans and people over agricultural resources (i.e., crop raiding, which sometimes results in the killing of orangutans), and the role of hunting of orangutans for meat, pets, and other reasons.

We aimed to answer the following research questions: (1) What are the demographic characteristics of the sampled human population?; (2) When and where have orangutans been sighted?; (3) What is the level of reported agricultural conflict with orangutans and how is this related to social and ecological variables?; (4) What is the level of reported killing of orangutans and how is this related to social and ecological variables?; (5) What is the nature of knowledge of Indonesian and customary (indigenous) law among survey respondents, and how is this related to reported killing?; and (6) What are the overall estimated killing rates, based on the survey data?

## Methods

### Ethics statement

The interview survey approach was reviewed and approved by the Nature Conservancy social science specialist. Participants in the surveys were informed of the goal of the interviews and assured that the data would be analysed anonymously.

### Survey design

**Figure 1:**
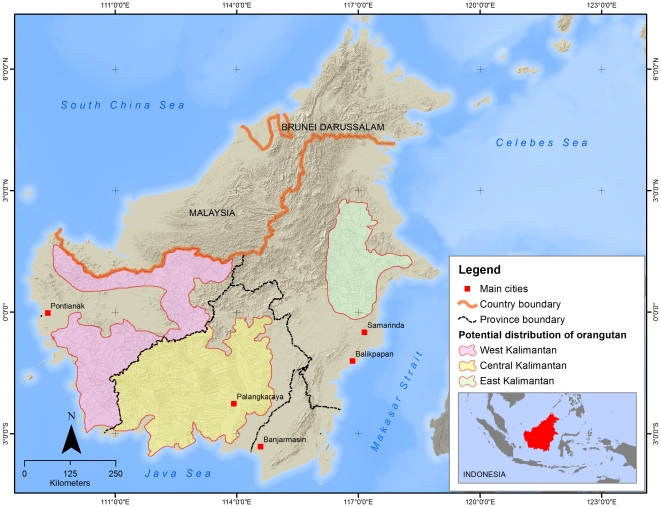
Potential distribution area of orangutan in Central Kalimantan (yellow), West Kalimantan (purple) and East Kalimantan (green), and the village area boundaries within these regions.

The survey was conducted in the 15-month period of April 2008 to September 2009, and covered three provinces in Kalimantan, where orangutans were known to occur: West, Central and East Kalimantan. Data were analysed over the seven months following the last survey. The survey involved collaboration by 18 conservation NGOs and was managed by The Nature Conservancy (TNC), the Association of Indonesian Primatologists (PERHAPPPI) and the Directorate of Forest Protection and Nature Conservation (PHKA), Ministry of Forestry. The survey area encompassed all regions with suspected orangutan presence, excluding specified areas (national parks) for which some information on hunting and human-orangutan conflict already existed. We sampled at the village level, as defined by the Indonesian Government Regulation No. 72 of 2005, which excludes cities. From the 4200 villages in all of Kalimantan, 1717 villages were identified that occur within the orangutan's distribution range (Figure 1). The distribution range was estimated by taking the 2004 Population and Habitat Viability Assessment (PHVA) distribution range [14] and buffering it with a 5 km zone around the range periphery [15]. Of these 1717 villages, 40% were selected as a stratified random sample (across high/medium/low risks of land use change), using an online random generator (http://www.randomizer.org/), resulting in a final selection of 687 villages.

The survey design was based on a questionnaire delivered to 10 residents in each of the villages surveyed (except in a small number of villagers where fewer than 10 respondents were available). Prior to the survey, information was collected about the village (age of village, number of families, number of schools, percentages of main religions, and main agricultural activities). The survey questionnaire comprised 32 questions and 34 optional sub-questions that were divided into a number of sections focusing on basic information (e.g., age, sex, religion, ethnicity of respondent), assessment of interviewee reliability (see below), questions on perceptions and experiences relating to orangutans (including orangutan encounters, occurrence of crop raiding, and killing), knowledge of national and customary laws, and forest use (e.g., reasons for entering forests, frequencies and length of forest trips). The detailed questionnaires are available in [15].

### Caveats of interview-based surveys

As in other disciplines, problems with biases and data quality may arise at any of the three main phases of an interview survey. At the design stage, questions can be raised about the sampling frame and sampling scheme (did the people we talked to accurately represent the broader target population?), the questionnaire (were questions well posed or potentially leading?), and the survey preparation (was a pilot study conducted, and how well were interview teams prepared?). At the data acquisition stage, key potential issues relate to data collection (e.g., were the data recorded faithfully?), respondent selection (e.g., did non-respondents differ meaningfully from respondents, or did respondents selected in different ways differ in their responses?), respondent reliability (could they reliably identify the focal species?) and respondent recall (did respondents give consistent replies about an event?). Finally, at the analysis stage, care must be taken to assess data quality, develop appropriate metrics, deal with missing data, choose appropriate statistical methods and models for estimation and inference, assess the sensitivity of the results and inferences, and produce accurate estimates of population-level variables, based on the sample statistics. In the implementation of this survey, we gave all these potential sources of bias considerable thought, and we have argued elsewhere that based on these precautions we consider our data a reliable reflection of reality (see [15], for a detailed description of the survey methodology).

### Spatial Analysis

To better understand the physical and geopolitical factors that influence orangutan killing, we compared the locations of reported killings and agricultural conflicts with a range of environmental factors (e.g., altitude, vegetation cover, rivers), and land use (e.g., timber concessions, oil palm concessions). We used GIS Software ArcGIS 9.3 (ESRI 2009) to measure the nearest (Euclidean) distances from the GPS points recorded for each village in the survey, to the boundaries of specified concession types (oil palm plantation, logging concession, timber estate) and protected forest (including national parks and nature reserves). Elevation and distance to the nearest river system were also calculated. The boundaries of logging concessions and timber estates were obtained from production forest distribution maps [16] and oil palm plantation boundaries were obtained from data provided by the Ministry of Agriculture [17]. Data for protected forest were obtained from provincial land use maps. We tested for spatial autocorrelation (Moran's I Index  =  −0.01, Z  =  −0.8), suggesting that spatial autocorrelation between the variables was not statistically significant at the 5% level.

### Missing data

The interviews resulted in many missing data, both at the individual level and village level. We recoded some variables to obtain the maximum number of full records. This included changing answers which were coded ‘0’ or ‘na’ (not available) to a shared code meaning that no information was available. For example, when the question about the occurrence of agricultural conflicts (1 = yes; 2 = no; 3 = don't know) was answered ‘0’ or ‘na’, we changed it to ‘don't know’. We also used contextual information to change codes or fill in blanks. For example, if no answer was available to the question whether the respondent had ever killed an orangutan, but that respondent had earlier replied to never having seen one, then we assumed that the respondent had not killed an orangutan.

### Respondent reliability

The ability of a villager to reliably identify an orangutan depends on a number of factors, including having ever seen one, familiarity with other similar species, access to televised broadcasts, and so on. In this study, the reliability of a villager's responses about orangutans was determined by asking respondents to identify 9 mammal species in a set of photographs, including several locally occurring primate species: orangutan, red langur (*Presbytis rubicunda*, a primate of similar colour as orangutan) and Bornean gibbon (*Hylobates* sp.).

Analyses of issues related to orangutans were restricted to respondents who were considered to be able to reliably differentiate an orangutan from similar species, i.e., the respondent correctly identified orangutan, and also correctly identified either or both the red langur and Bornean gibbon. If the respondent failed to recognize the orangutan, or only claimed to know the orangutan but neither of the other species, he or she was classed as unreliable.

Respondent reliability was also assessed by cross-validation of responses (e.g., ever have seen an orangutan and having seen one in the past year). Records with incompatible responses were omitted from the corresponding analyses.

### Statistical Methods

Two statistical approaches were used to analyse the questionnaire data. The first approach was an analysis at the village level, using multiple general and generalized linear regression models (GLM). The second approach was an analysis at the respondent level, using general and generalized linear mixed models (GLMM) in which individual responses were nested within villages, which were, in turn, nested within relevant districts/regions. Binary and multinomial responses were fitted using a logistic regression model with all explanatory variables entered. Model fit was evaluated by the deviance and likelihood ratio chi-square tests; the goodness of fit of all the reported models was statistically significant based on this test. Results of the regression analyses are reported as relative probabilities and relative risks.

The respondent-level factors in the GLM and GLMM models for analysis of conflict included age, sex and tribe group. The respondent-level factors in the models for analysis of killing included age, sex, tribe group, knowledge of customary law, knowledge of Indonesian law, total trips to the forest and primary reason for entering the forest.

The village-level factors in the GLM models included population size for each village (answered in two ways, as number of families or number of people), dominant religion for each village (based on interview data), size of a village area, population density for each village (population size per size of village area), and dominant professional occupation (based on interview data). The village-level factors in the GLMM models included number of individuals in the village, proportion of the village that was Muslim, Christian or of other religion, number of schools per family and presence/absence of the following agricultural or other businesses: oil palm, coconut, rice, rubber, cacao, pepper, vegetables, fishing, and industrial plantations or mines.

The spatial data were also analysed using generalized linear (logistic) models. Models were fitted with and without interactions.

### Estimation of total killing rates

A range of estimates were obtained for the total killing rate in Kalimantan. The values were based on two survey questions, the first regarding the total number of animals killed by the reliable participants themselves and the second regarding the total number of animals killed in the village area in the last year. Analyses were confined to reliable respondents, after excluding obvious outliers (see Results).

Two individual-based estimates were considered: (i) the total number killed by each respondent; (ii) the average number killed per year by each respondent, which was calculated as (total number killed by respondent/respondent's age minus 13—the minimum age of respondents who reported killing an orangutan, and consistent with census age groups).

Since respondents in a village may have all reported killing(s) of the same animal(s) or different animals, two village-based estimates were considered: (i) the number reported killed in the village in the last year, summed over all respondents in the village, and (ii) the average number reported killed in the village in the last year. The first of these estimates assumes that all respondents reported about different killings and is therefore a liberal estimate of the annual killings. The second value assumes equal weighting for each villager, and is thus a conservative estimate; i.e., all villagers have equal knowledge about all orangutan killings in the village area. Based on our experience of Kalimantan villages, the extent to which killings become widely known in a village primarily depends on the size of the village (in large villages, an orangutan killing may go largely unnoticed), and also extent of social cohesion. Because we lacked empirical data on these factors, we did not use a village-size based correction factor.

The estimates developed above from the sampled reliable participants were extrapolated to the entire sampling frame; see [15] for details. In brief, the survey comprised a 40% sample of the 1717 villages in the sampling frame (the area hypothesised to encompass the full range of orangutan in Kalimantan; 558 villages in West Kalimantan, 976 in Central Kalimantan, 183 in East Kalimantan), stratified by high/medium/low threat of land-use change. Hence the sampling weights were based on the threat stratification and province.

For the individual-based killing rates, the sample estimates (based on 6983 respondents in 687 villages) were extrapolated to the target population in each province using weights based on the available census data for 2006 and 2008 from the Indonesian Bureau of Statistics. Since only 8 women reported killing orangutans, the target population was taken to be males aged 15 years and older. Based on the census data, 71.5% of the population was aged 15+ years (giving 557867 people in the Central Kalimantan part of the orangutan distribution range, 39620 in East Kalimantan, 234058 in West Kalimantan) and the sex ratio (male/female) was 1.09. These figures were then multiplied by 1717/687 and a further adjustment was made to account for reliable respondents. Finally, a finite population correction factor was applied to account for the large sample: √((N-n)/(N-1)) where n is the sample size and N is the population size. The uncertainty associated with the four estimates was also calculated and expressed as 95% confidence intervals.

A number of statistical packages were used for the analyses. These included SPSS and SAS for the summary analyses, and MLWin for the multilevel analyses, which were fit as generalized linear mixed models. For all analyses, statistical significance was indicated by p-values of ≤0.05.

## Results

### Respondents' demography and orangutan sightings

In terms of demographics, 89% of all surveyed respondents were male and 11% were female. The majority of respondents were classified as of Dayak origin (66%; Dayak is a collective name for indigenous ethnic groups, mostly from the interior of Borneo), followed by Malay, Banjar and Kutai people who are predominantly coastal (17%), immigrants (17%; consisting of Javanese, Balinese, Buginese and others), and formerly nomadic people (<1%; Punan and Orang Ut). Of all respondents, 2% had resided in the present village for less than 2 years and 12% for less than 10 years. 71% had resided there for at least 20 years and 27% for at least 40 years. The respondents were mainly Muslims (45%) and Christians (44%), with the Keharingan religion (which has similarities with Hinduism) an important third group, especially in Central Kalimantan.

Based on the photos of orangutans and similar looking primate species, 17% of all respondents could not reliably identify an orangutan. 13% could identify an orangutan and at least one of the other two selected species, and 70% could identify all three selected species. When categorized by ethnicity, immigrants had the largest proportion of unreliable respondents (37%), followed by Malay, Banjar and Kutai people (15%), Dayaks (13%) and Punans (<0.1%).

Among reliable respondents, 42% (n = 2086) reported that they had seen orangutans around the village. Over half of this group (56%) said that the last sighting was more than a year ago, while 20% reported seeing one in the last month. This subgroup reported that orangutans were usually seen in the forest (78%), gardens/farms (8%), on the road (2%) and in other locations (12%). Similar percentages were obtained for reliable respondents who had seen an orangutan in the last year. The demographics of the subgroup of reliable respondents that had seen an orangutan around the village were similar to the demographics of the whole sample, with respect to gender (93% (n = 1935) male, 7% (n = 151) female), tribal group (67% Malay, 19% coastal, 13% immigant, <1% formerly nomadic), duration of residence (1.4% <2 years, 7.6% >10 years, 78% >20 years, 31% >40 years) and religion (45% Muslim, 41% Christian, 14% other).

### Conflict

**Figure 2:**
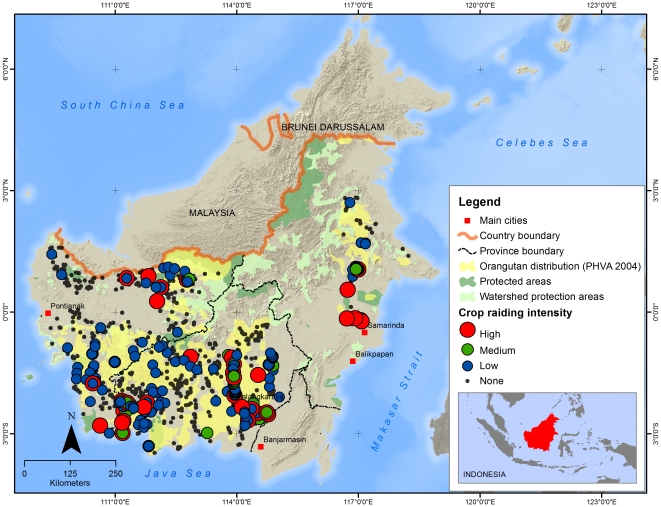
Crop raiding intensity in different villages across Kalimantan. High  =  reported conflict frequency every week; Medium  =  every month; Low  =  once a year or less frequently.

Among reliable respondents who had seen an orangutan around the village, 15% reported that agricultural conflicts with orangutans had occurred at some time in their village. Of these respondents, 33% said this conflict had occurred frequently (every week or month), 20% once a year, and 47% rarely. The time period over which these frequencies applied was not assessed. These figures varied slightly among provinces. East Kalimantan had the highest level of any conflict (18% of reliable respondents reporting conflict) and of frequent conflict (8%). Corresponding figures were 15% and 5% for Central Kalimantan, and 12% and 1% for West Kalimantan. Crop raiding by orangutans was thus reported throughout Kalimantan, but it seems particularly severe in central East Kalimantan, eastern and western Central Kalimantan and southern West Kalimantan (Figure 2).

Respondents expressed a range of different reactions to orangutans that entered their gardens. Among reliable respondents who had seen an orangutan around the village and experienced conflict, almost half (46%) reacted by attempting to chase or scare the orangutan away, but 5% reacted by killing or trying to kill it. The remainder (49%) reported ‘other’ reactions. Similar percentages were found among those who had experienced conflict within the last year.

### Regression analyses of conflict

The regression analyses showed that reliable respondents are more likely to report crop raiding if the village area encompasses production areas for palm oil, rice or industrial pulp and paper plantations (p<0.1, 0.05, 0.001 respectively). Some differences were also found between tribal groups. No other respondent-level nor village-level variables were significantly associated with crop raiding intensity.

For reliable respondents who had seen an orangutan around the village, the GIS data analyses revealed that the relative risk of any conflict significantly increased at lower elevations (p<0.001), with increasing distance from a river (p<0.05), with increasing proximity to the nearest logging concession (p<0.000), and with increasing distance from the nearest pulp and paper plantation (p<0.001) or from protected forest (p<0.001). This suggests that agricultural conflicts mostly occur in Kalimantan's lowlands, away from rivers, where most people live, and where orangutan habitat fragments are most likely to remain. This general picture changes at higher elevations, where higher agricultural conflict rates were observed closer to a river at higher elevations and close to a logging concession.

### Killing

At the village level, in 179 villages out of 687 (26%) at least one reliable respondent reported that at least one orangutan had been killed at some time. In 145 villages out of 687 (21%) at least one respondent reported to have killed an orangutan. Among reliable respondents who had seen an orangutan around their village, 42% reported that an orangutan had been killed in the village at some time; 8% reported that one had been killed in the past year, 12% in the past five years and 22% more than five years ago. Among those who provided the number of orangutans that had been killed, most (73%) reported 1 animal killed, 24% reported 2 or 3 killed, and 4% reported more than three.

Among all reliable respondents who responded to the question of whether they had killed an orangutan, 232 out of 4732 (4.9%) responded ‘yes’. Of these, 2.7% (n = 127) reported that they had killed 1 orangutan; 1.3% (n = 80) reported killing 2; 0.5% (n = 23) respondents reported killing between 3 and 20 orangutans; and two reported killing respectively 70 and 100 animals. Among all reliable respondents who reported that they had ever seen an orangutan around the village and who responded to the question of whether they had killed an orangutan, 160 out of 1511 (9.6%) responded ‘yes’. Of these, 4.7% (n = 79) reported that they had killed 1 orangutan, 2.9% (n = 49) reported killing 2; 31 respondents reported killing 3–20 orangutans; and one reported killing 100 animals (total killed by respondents  = 629 orangutans). Details of the interview with this last respondent were published elsewhere [18], suggesting that such high kills rates were restricted to hunters that specialised in killing orangutans.

**Figure 3:**
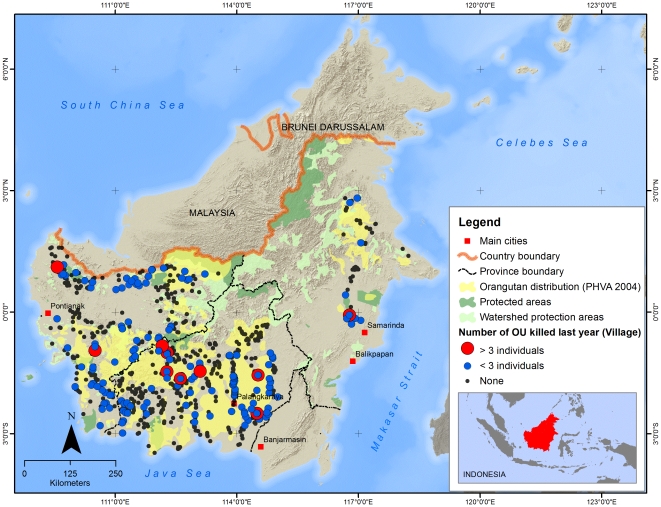
Reported orangutan killing in 2008–2009 in three provinces of Kalimantan.

The killing rates differed between the three provinces. The percentages for numbers of orangutans killed were highest in Central Kalimantan and lowest in West Kalimantan (Figure 3). The killing rate figures were lower when analysis was restricted to reliable respondents who had seen an orangutan in the last year. In this group, 26% reported that an orangutan had been killed in the village, with 4% in the past year, 7% in the past five years and 15% more than five years ago. The distribution of number of orangutans killed was the same. One in twenty (5%) reported that they themselves had killed an orangutan; 3% reported killing one orangutan, 2% reported killing 2–5, and less than 1% reported killing between 6 and 100 orangutans.

Reported reasons for orangutan killings occurring in the village (i.e., not necessarily involving the respondent), were primarily for food (54%), self-defence (14%), don't know (11%), pest of crops (10%) and other reasons (combined 11%). Very small proportions of respondents reported that killing occurred for traditional medicine, to sell baby orangutans, hunting for fun or being paid to kill. Notably, those who reported they had personally killed (an) orangutan(s) did so for an “unknown reason” (41%), and only 14% said they did so for food, 7% for self defence or because the orangutan was perceived as a pest, while others cited other reasons (38%).

### Regression analyses of killing

Regression analyses of reliable respondents who had seen an orangutan around the village revealed that religion, total time spent in the forest and reason for entering the forest were significantly associated with the probability of reported killing of an orangutan. The age, sex, and ethnic background of respondents and the existence in their village of customary laws protecting orangutans were not significantly associated with killing an orangutan. The probability of reportedly killing orangutans increases significantly with increasing time spent in the forest (p = 0.0001). Compared with those who entered the forest primarily for gathering non-timber forest products or other reasons, the probability of killing orangutans was significantly higher for respondents who entered the forest primarily for logging, hunting or mining (compared to non-timber forest products; p<0.01).

As opposed to the occurrence of agricultural conflicts, which our geographical analysis suggested to primarily occur in lowland areas, possibly along the forest transition boundary, orangutan hunting is more concentrated in the forested upland parts of Kalimantan. Among reliable respondents who had seen an orangutan around the village, the relative risk of reported killing of an orangutan by a respondent increased with increasing elevation (p<0.01), increasing distance from a river (p<0.001) or forest patch (p<0.01), and increasing proximity to a logging concession (p<0.01). Although the relative risks associated with distance to a palm oil plantation or logging concession were not significant overall, they were significantly higher at higher elevations (p<0.01). Similarly, the risk associated with distance to a protected forest was higher at higher elevations (p<0.05). The killing rates associated with proximity to an oil palm plantation, logging concession or protected forest were doubled among villages at high elevations, compared with low elevations.

### Overall killing rates

Estimates of the overall rate of killing of orangutans were as follows. Based on the reported number of orangutans killed by reliable individual respondents at any time in their lives, the total number of orangutans killed per year is 2540 (95% CI 1970-3100). Based on the total number of orangutans reportedly killed around the villages in the last year, the estimated total number of orangutans killed in the last year is 1750 (95% CI 1700-1790). Based on the average of the responses in each village, the estimated total killing rate in the last year is 785 (95% CI 750-815). We excluded the above mentioned outliers of >20 reported/kills per respondent from all these extrapolations.

These results give rise to two ranges: between 750 and 1790 for the number of orangutan killed in the last year; between 1970 and 3100 for the average number of orangutan killed per year in the orangutan's 2004 distribution range in Kalimantan within the lifetime of the respondents. It is acknowledged that the killing rate and the population rate (and spatial distribution) have changed over the years, and that these cannot be definitively estimated based on the questionnaire data. However, it is still valid to state an average rate. This also implies that if the number killed per year in recent years is larger, and if the number of orangutans has decreased in this period, then the relative killing rate has increased, and has been higher in recent years than our estimated average.

### Correlation between conflict and killing

There was a highly significant association between reported conflict and killing. Among reliable respondents who had encountered an orangutan around the village, 23% (n = 53/231) of those who reported conflict also reported personally killing an orangutan, compared with a killing rate of 7% (n = 107/1440) among respondents who reported no conflict (χ^2^ = 55, df = 1, p<0.001). There was no significant difference between killing rates associated with frequency of conflict: (21% for conflict every week or month, 24% for conflict once a year or less) (χ^2^<1, p>0.05). The same trends were observed among all reliable respondents, with reported killing rates of 4% (n = 167/4249), 20% (n = 42/171) and 23% (n = 23/79) associated with no conflict, less frequent conflict and more frequent conflict, respectively. There was also a significant association between killing rates and response to conflict: 53% (n = 10/19) of the respondents who reported trying to kill an orangutan who entered their garden also reporting having killed an orangutan, compared with a killing rate of 28% (n = 56/303) among those who reported other responses (scare away, throw, other) (χ^2^ = 12, df = 1, p<0.001). There was no association between response to conflict (try to kill; other) and reason for killing an orangutan (pest or defence; other). Further inferences about the nature of the relationship between reported conflict and killing are difficult, since the numbers of respondents in these analyses are small and the questions did not allow substantive causal relationships to be drawn.

### Knowledge of Indonesian and customary law (indigenous law)

Customary law is the local indigenous law that may or may not exist in different tribes, whereas Indonesian law is the national law stipulating that orangutans are fully protected. In our survey, among all individuals who responded to the relevant questions (n = 6972), 15% reported that orangutans were protected under their customary laws, 53% reported that they were not protected, and 32% reported that they did not know. Most people (73%) responded that they knew that orangutans were protected under Indonesia's national law, 2% reported that they were not protected and 25% reported that they did not know. There was a highly significant association between responses about orangutan customary law and national law. Of those who reported that orangutan are protected by national law, 19% replied that orangutans are also protected by customary law; 53% replied that the species is not protected by customary law, and 28% replied that they did not know about orangutan customary law. Of those who reported that orangutans are not protected by national law, 74% reported that orangutans are also not protected by customary law and 23% reported that they do not know.

Knowledge of national law differed significantly among different ethnic groups. A substantially smaller proportion of Dayak groups, and larger proportion of immigrant people, reportedly knew that orangutans are protected under the laws of the Republic of Indonesia. The proportion of respondents who reportedly did not know about national law for orangutan was largest for Punans, followed by Dayak groups, and smallest for immigrants.

Although knowledge of customary law was not significantly associated with reported personal killing of an orangutan when considered in conjunction with other variables, the two variables were highly significantly associated (p<0.001) when considered alone. Among reliable respondents who had encountered an orangutan around the village, the killing rate was much higher among those who reported that orangutans were protected by customary law (15%) compared with those who reported that they were not (10%) and those who did not know (7%). This suggests that customary laws provide little protection to orangutans, although we note that the associations do not necessarily imply causal relationships. In contrast, in the same group of respondents, the killing rate was much lower among those who reported that orangutans were protected by Indonesian law (8%) compared with those who reported that they were not (19%) and those who did not know (13%).

## Discussion

Limitations of the study results arising from potential biases have been addressed by Meijaard et al. [15], but we briefly reiterate the main points. Possible bias due to excluding unreliable responses was shown to be insubstantial. Interviewer bias was also shown to be controlled. Interviewer bias was controlled by training and the possibility of interviewers inventing data was taken into account. Respondent bias was addressed in four ways, including analysis of the precision of responses regarding the location of reported sightings, comparison of responses within and between villages, evaluation of responses to overlapping questions with different timeframes and variation in villagers' responses within villages to questions about orangutans. With regard to the last of these, there was greater consistency in responses that no orangutan had being killed in the village than claims about killing. While some of this inconsistency was related to age and length of residence in the village, and while it may also point to lack of information flow (i.e. the killing of an orangutan does not become known to everyone), it may alternatively imply that killing is misreported by individual respondents. This social desirability bias may be negative (leading to under-reporting), possibly due to knowledge that killing of orangutans is illegal, or positive (leading to over-reporting) if respondents are inclined to boast about killing or if they perceive that positive responses are related to good hunting skills or knowledge of the forest. We are also aware of incomplete recall over longer time frames [19], [20], but think that our confidence intervals and two different approaches to estimating killing rates sufficiently capture this bias. As with all surveys, it is difficult to quantify the possible magnitude of this bias overall, let alone disentangle the contributions of the direction of the bias. Further evaluation would require a follow-up validation survey, which was not undertaken in this study.

Our study suggests that although only a small proportion of respondents reported that human-orangutan conflict exists, the implication of this conflict is important. There is scientific consensus that orangutan population decline has mostly been associated with the loss of habitat [8], [21], [22], [23]. While this is true, so far we have not really addressed the implication of the loss of habitat and the potential conflict that results. Our data suggest that conversion of forests to other land uses can result in orangutans entering villagers' gardens and raiding crops. This form of direct conflict can result in killing. Killing can also arise for other reasons, including hunting for food, or more rarely to obtain orangutan babies for the pet trade, to use orangutan parts for medicinal purpose, because people were scared, or because people were paid to hunt orangutans (this was rarely reported, but respondents may have been more reluctant to disclose this, and its prevalence remains unknown). In this study, killing incidence was highest among villagers who regularly entered the forest for logging or hunting. This finding has important implications for policymakers and law enforcements. Awareness raising for orangutan conservation needs to be targeted in general at communities that live in close proximity to orangutan habitat, and especially those groups that are most likely to hunt orangutans.

Our results showed that respondents are more likely to report crop raiding if the village area encompasses production areas of palm oil, rice or industrial forest plantations. The highest rates of conflict were reported in East Kalimantan Province. Although this province has a higher total forest cover compared to the other two provinces, high rates of conflict occur in an area that was mostly deforested in the 1980s, but still contains orangutans in a matrix of pulp and paper as well as oil palm plantations and a few remaining stands of degraded forest [24]. More detailed work is needed to determine exactly in which areas the killings and conflicts occur; there are reports of high killing rates in several oil palm plantations in the region (Y. Rayadin, pers. comm. to EM). Across the provinces, the incidence of killings is highest in Central Kalimantan, a province that has the largest population of orangutans [25], and high rates of land-use change from forest to agriculture [26]. Overall, our data suggests that human-orangutan conflict and killings are most prominent in south-eastern and south-western Central Kalimantan, southern and northern West Kalimantan, and a small area in East Kalimantan. Northern West Kalimantan is generally an area with high deforestation rates and rapid plantation development, and especially in the part of the province which was once an area of very high orangutan densities [9], very little natural forest habitat remains. It is likely that the few remaining orangutans often encounter people, thus leading to high killing and conflict rates.

The estimated annual and total killing rates of orangutans and their spatial patterns are highly worrying. Orangutans are killed throughout Kalimantan in numbers that appear far above maximum take off rates for viable populations. Population viability studies of orangutans suggest that if annual mortality of females is higher than 1% then populations will go extinct [27]. We do not have data that distinguish between killings of male or female orangutans, but we assume that the ratio is about 1∶1. It could be biased towards females, which are smaller than males and often accompanied by juvenile orangutans, a possible target for the pet trade, but it could also be biased towards unflanged males which are more likely to leave their natal population and roam widely. This would suggest that between 375 and 1550 females were killed in the year prior to the study. On a total population of some 42500 animals in Kalimantan [25], this would imply annual female take off rates between 0.9 and 3.6%. These mortality rates caused by hunting alone are higher than the theoretical maximum mortality for population viability, suggesting that unless they can be reduced most Kalimantan populations will go extinct. The potential biases mentioned at the start of the Discussion may impact on the estimated conflict and killing rates. Although the magnitude of the under- or over-estimation is unknown, based on the analyses undertaken in this paper and in [15], we argue that the relative size of this uncertainty is small compared with the uncertainty already incorporated in the reported ranges for the estimates of the number of orangutans killed in the last year and the number killed per year.

The spatial analysis of conflict and killing suggests complex relationships. Overall, conflict and killing occur throughout Kalimantan, but with hotspots in particular areas. These hotspots seem to be influenced by land use and proximity to forest, with the highest killing rates in relatively intact forests at higher altitude. How these patterns interact with socio-cultural factors remains unclear, but the data suggest that no orangutans outside Kalimantan's protected areas are safe. They are either threatened by habitat degradation and deforestation, or they are threatened by ongoing hunting within their forest habitats. Unless effective countermeasures are implemented, we anticipate further killings in the near future. There is a great urgency to address this situation strategically.

To develop effective awareness and law enforcement campaigns, we need to understand the reasons for the killings and the demographic and socio-ecological characteristics of the people who kill orangutans. This is important, because, despite evidence that social factors are important in human-wildlife conflict, they are often ignored in conflict studies [7]. Our data show a conflicting result between perceived reasons for killing an orangutan in general and reasons for personally killing an orangutan. While the primary general reason that respondents gave for killings (in general) was for food, the reasons given by individuals who had killed orangutan themselves were quite varied, ranging from accidents, tree fell (because of logging), caught in traps, source of food, etc. These differences may arise because people who killed an orangutan may know that the species is protected by law; hence their answer became ambiguous when they were asked directly about this issue. Follow up surveys should consider the use of complementary methods such as randomized response technique (RRT), designed for investigating sensitive behaviours (e.g., [28]).

Our results show that killing is associated with ethnicity, religion, reason for entering the forest, and perception of the orangutan as a pest or personal threat. This suggests that anti-killing measures could be targeted to specific groups with specific messages and approaches. For example, if religious affinity is an important factor it might be an idea to channel messages on conservation law and ethics through religious institutions. Importantly, even though many orangutans appear to be killed every year, most people never kill one, and most that do have killed only one or a few orangutans in their lifetimes. This suggest that most people who kill may do so opportunistically, and it might be relatively easy to convince people that such killings are no longer socially acceptable. Targeting hunting through media campaigns might be effective because it may not take that much to stop people from making those kills.

Our results also show that when other factors were taken into account, knowledge of customary law was not significantly associated with killing an orangutan, meaning that the existence of the law and the knowledge of its existence is not effective in protecting orangutan, even though in the past such taboos appear to have been stronger [8]. This conclusion was supported by independent analysis of killing and knowledge of customary law: the killing rate was found to be 50% higher among those who reported that such a law existed, compared with those who reported that such a law did not exist. In contrast, knowledge of Indonesian law was associated with lower killing rates: those who knew of the law reported a killing rate less than half that of those who said they did not know of this law. Note that this observation does not of itself imply a causal link between knowledge of laws and killing rates.

The overall message of our result is that killing is a major threat to orangutans in Kalimantan. This threat adds to habitat loss, the other main cause of orangutan population decline. Like other human wildlife conflict [29], [30], we need to address this situation institutionally and legally and at the same time work directly with the communities and land managers to stop the killings. Importantly, our study has revealed important spatial, demographic and socio-ecological factors associated with killing that can be used to target this work more effectively.

## References

[1] McShane TO, Hirsch PD, Trung TC, Songorwa AN, Kinzig A (2011). Hard choices: Making trade-offs between biodiversity conservation and human well-being.. Biol Conserv.

[2] Hoffmann M, Hilton-Taylor C, Angulo A, Bohm M, Brooks TM (2010). The impact of conservation on the status of the world's vertebrates.. Science.

[3] Leadley P, Pereira HM, Alkemade R, Fernandez-Manjarrés JF, Proença V (2010). Biodiversity Scenarios: Projections of 21st century change in biodiversity and associated ecosystem services.. CBD Technical Series no. 50.

[4] Campbell-Smith G, Simanjorang HVP, Leader-Williams N, Linkie M (2010). Local attitudes and perceptions toward crop-raiding by Orangutans (*Pongo abelii*) and other nonhuman primates in Northern Sumatra, Indonesia.. Amer J Primat.

[5] Hartter J, Goldman A, Southworth J (2010). Responses by households to resource scarcity and human-wildlife conflict: Issues of fortress conservation and the surrounding agricultural landscape.. J Nat Conserv.

[6] Nijman V, Nekaris KAI (2010). Effects of deforestation on attitudes and levels of tolerance towards commensal primates (Cercopithecidae) in Sri Lanka.. Int J Pest Manage.

[7] Dickman AJ (2010). Complexities of conflict: the importance of considering social factors for effectively resolving human-wildlife conflict.. Anim Conserv.

[8] Rijksen HD, Meijaard E (1999). Our vanishing relative.. The status of wild orang-utans at the close of the twentieth century.

[9] Meijaard E, Welsh A, Ancrenaz M, Wich S, Nijman V (2010). Declining orangutan encounter rates from Wallace to the present suggest the species was once more abundant.. PloS ONE.

[10] Goossens B, Chikhi L, Ancrenaz M, Lackman-Ancrenaz I, Andau P (2006). Genetic signature of anthropogenic population collapse in orang-utans - art. no. e25.. PLoS Biol.

[11] Hockings K, Humle T (2009). Best Practice Guidelines for the Prevention and Mitigation of Conflict between Humans and Great Apes..

[12] Campbell-Smith G, Campbell-Smith M, Singleton I, Linkie M (2011). Raiders of the lost bark: Orangutan foraging strategies in a degraded landscape.. PloS ONE 6.

[13] IUCN (2010). http://www.iucnredlist.org.

[14] Singleton I, Wich SA, Husson S, Atmoko SU, Leighton M (2004). Orangutan Population and Habitat Viability Assessment: Final Report..

[15] Meijaard E, Mengersen K, Buchori D, Nurcahyo A, Ancrenaz M (2011). Why don't we ask? A complementary method for assessing the status of great apes.. PloS ONE.

[16] The Ministry of Forestry Republic of Indonesia (2010). Map of Distribution IUPHHK HA/HT/HTR (September 2010) in The 3rd Quarter Progress Report of Production Forest Management and Utilization..

[17] The Ministry of Agriculture Republic of Indonesia (2010). Map of Distribution of Oil Palm Plantation..

[18] Nurcahyo A (2009). The two different monkey chasers.. Forest Sc News.

[19] Neter J, Waksberg J (1964). A study of response errors in expenditures data from household interviews.. J Amer Stat Assoc.

[20] Beaman J, Vaske JJ, Miller CA (2005). Cognitive processes in hunters' recall of participation and harvest estimates.. J Wildl Manag.

[21] Ancrenaz M, Ambu L, Sunjoto I, Ahmad E, Manokaran K (2010). Recent surveys in the forests of Ulu Segama Malua, Sabah, Malaysia, show that orang-utans (*P. p. morio*) can be maintained in slightly logged forests.. PLoS ONE.

[22] Husson SJ, Wich SA, Marshall AJ, Dennis RA, Ancrenaz M, Wich SA, Atmoko SU, Setia TM, van Schaik CP (2009). Orangutan distribution, density, abundance and impacts of disturbance.. Orangutans: geographic variation in behavioral ecology and conservation.

[23] Wich SA, Singleton I, Utami-Atmoko SS, Geurts ML, Rijksen HD (2003). The status of the Sumatran orang-utan *Pongo abelii*: an update.. Oryx.

[24] Meijaard E, Albar G, Rayadin Y, Nardiyono, Ancrenaz M (2010). Unexpected ecological resilience in Bornean Orangutans and implications for pulp and paper plantation management.. PloS ONE.

[25] Wich SA, Meijaard E, Marshall AJ, Husson S, Ancrenaz M (2008). Distribution and conservation status of the orang-utan (*Pongo* spp.) on Borneo and Sumatra: how many remain?. Oryx.

[26] Miettinen J, Shi C, Liew SC (2011). Deforestation rates in insular Southeast Asia between 2000 and 2010.. Glob Change Biol.

[27] Marshall AJ, Lacy R, Ancrenaz M, Byers O, Husson S, Wich S, Atmoko SU, Mitra Setia T, van Schaik CP (2009). Orangutan population biology, life history, and conservation. Perspectives from population viability analysis models.. Orangutans: geographic variation in behavioral ecology and conservation.

[28] John FAV St, Keane AM, Edwards-Jones G, Jones L, Yarnell RW (2011). Identifying indicators of illegal behaviour: carnivore killing in human-managed landscapes.. Proc Roy Soc B: Biol Sc.

[29] Treves A, Karanth KU (2003). Human-carnivore conflict and perspectives on carnivore management worldwide.. Conserv Biol.

[30] Webber AD, Hill CM, Reynolds V (2007). Assessing the failure of a community-based human-wildlife conflict mitigation project in Budongo Forest Reserve, Uganda.. Oryx.

