# Correction: Quantifying Killing of Orangutans and Human-Orangutan Conflict in Kalimantan, Indonesia

**DOI:** 10.1371/annotation/e6ef5bb6-449c-458f-8e90-2629d91e1767

**Published:** 2012-03-06

**Authors:** Erik Meijaard, Damayanti Buchori, Yokyok Hadiprakarsa, Sri Suci Utami-Atmoko, Anton Nurcahyo, Albertus Tjiu, Didik Prasetyo, Nardiyono, Lenny Christie, Marc Ancrenaz, Firman Abadi, I Nyoman Gede Antoni, Dedy Armayadi, Adi Dinato, Ella, Pajar Gumelar, Tito P. Indrawan, Kussaritano, Cecep Munajat, C. Wawan Puji Priyono, Yadi Purwanto, Dewi Puspitasari, M. Syukur Wahyu Putra, Abdi Rahmat, Harri Ramadani, Jim Sammy, Dedi Siswanto, Muhammad Syamsuri, Noviar Andayani, Huanhuan Wu, Jessie Anne Wells, Kerrie Mengersen

This Note accompanies the Correction on this article and provides a link to a revised version of the article that incorporates the results obtained after the re-analysis of the dataset having excluded the duplicated data. The revised manuscript can be viewed here: http://www.plosone.org/corrections/pone.0027491.article_correction.cn.pdf

