# Correction: Quantifying Killing of Orangutans and Human-Orangutan Conflict in Kalimantan, Indonesia

**DOI:** 10.1371/annotation/7b65cf64-9fd5-4b95-baf2-8824c4785ab1

**Published:** 2012-03-06

**Authors:** Erik Meijaard, Damayanti Buchori, Yokyok Hadiprakarsa, Sri Suci Utami-Atmoko, Anton Nurcahyo, Albertus Tjiu, Didik Prasetyo, Nardiyono, Lenny Christie, Marc Ancrenaz, Firman Abadi, I Nyoman Gede Antoni, Dedy Armayadi, Adi Dinato, Ella, Pajar Gumelar, Tito P. Indrawan, Kussaritano, Cecep Munajat, C. Wawan Puji Priyono, Yadi Purwanto, Dewi Puspitasari, M. Syukur Wahyu Putra, Abdi Rahmat, Harri Ramadani, Jim Sammy, Dedi Siswanto, Muhammad Syamsuri, Noviar Andayani, Huanhuan Wu, Jessie Anne Wells, Kerrie Mengersen

Since our paper was published in PLoS ONE we have become aware of data duplication in our data set. We subsequently re-analyzed the data, leaving out those that had been inappropriately collected, and hereby present the revised findings. The overall conclusion that hunting and conflict killing is a major threat to orangutans in Kalimantan remains unchanged.

Our original data were collected by 19 different survey teams, and consisted of 6,983 cases with 82 text and numeric data entries each. When working on a previously unused part of the dataset we noticed some repeated text entries. One of the 19 survey teams had apparently copied data from 10 villages across to others villages (9 times a duplication, and once a triplication), and subsequently made small changes in the text, presumably to cover up the duplication.

This puts into doubt the veracity of 209 out of 6,983 cases (3%), or half that if we assume that interviews were actually conducted in one village before being duplicated to another village. We rechecked the dataset and could not find other indications of data duplication. To be on the safe side, we re-analyzed our dataset by excluding all 570 cases that the particular survey team had worked on, i.e., 8.1% of the total data. In this we assumed that if they duplicated part of the data, the reliability of their remaining data was also in doubt. The objective was to evaluate the impact that the potentially unreliable data had on our overall findings.

Leaving out 8.1% of the data changes many of the quantified responses provided in our publication. The important overall estimates of annual killing rates of orangutans, however, have only changed a little, and our general conclusions that killing of orangutans is a major threat to their survival remains unchanged. Estimated killing rates based on the original data set were between 750 and 1800 animals killed in the last year, and between 1950 and 3100 animals killed per year on average within the lifetime of the survey respondents. After re-analyzing the data set which excluded the potentially 570 fraudulent records, this changed to between 630 and 1357 animals killed in the last year, i.e. somewhat lower than the original estimate, and between 2383 and 3882 animals killed per year on average within the lifetime of the survey respondents, i.e. somewhat higher than the original estimate. We believe that the core message of the original paper—killing and conflict were a much higher threat to orangutans than previously thought—remains unchanged. In conservation terms, the magnitude of these kill rates is more important than the actual estimate, because it drives home the message that unless killings are seriously considered as a threat and strategies developed to abate this, most of Kalimantan's orangutans will be hunted out in the next few decades.

We always knew that data manipulation was a risk when working across such a vast area with many different teams and limited supervision. We addressed this issue on page 9 of the methodological paper that we published in PLoS ONE prior to the present one. We quote: “Another issue of concern is the extent to which interview teams could make up the data rather than obtain actual interviewee responses”. In our methodological paper we clarified how we used 4 approaches to minimize chances of fraud, but we also admit that we could not fully exclude it. Our present findings unfortunately confirm that we were right about that.

In conclusion, we regret that the data duplication has occurred and was not picked up prior to publication of our paper. We maintain however that our original findings presented in that paper are still supported following re-analysis of the dataset as shown in the revised manuscript.

